# Transfer of AgNPs’ Anti-Biofilm Activity into the Nontoxic Polymer Matrix

**DOI:** 10.3390/polym15051238

**Published:** 2023-02-28

**Authors:** Lívia Mačák, Oksana Velgosova, Erika Múdra, Marek Vojtko, Silvia Dolinská

**Affiliations:** 1 Institute of Materials and Quality Engineering, Faculty of Materials Metallurgy and Recycling, Technical University of Kosice, Letná 9/A, 042 00 Košice, Slovakia; 2 Division of Ceramic and Non-Metallic Systems, Institute of Materials Research, Slovak Academy of Sciences, Watsonova 47, 040 01 Košice, Slovakia; 3 Institute of Geotechnics, Slovak Academy of Sciences, Watsonova 45, 040 01 Košice, Slovakia

**Keywords:** silver nanoparticles, green synthesis, *Lavandula angustifolia*, TEM, SEM, PVA matrix, polymer composite

## Abstract

A biological method was successfully applied to synthesize spherical silver nanoparticles (AgNPs) while using the extract of lavender (Ex-L) (lat. *Lavandula angustifolia*) as the reducing and stabilizing agent. The produced nanoparticles were spherical with an average size of 20 nm. The AgNPs’ synthesis rate confirmed the extract’s excellent ability to reduce silver nanoparticles from the AgNO_3_ solution. The presence of good stabilizing agents was confirmed by the excellent stability of the extract. Nanoparticles’ shapes and sizes did not change. UV-Vis absorption spectrometry, Fourier transform infrared spectroscopy (FTIR), transmission electron microscopy (TEM), and scanning electron microscopy (SEM) were used to characterize the silver nanoparticles. The silver nanoparticles were incorporated into the PVA polymer matrix by the “ex situ” method. The polymer matrix composite with AgNPs was prepared in two ways: as a composite film and nanofibers (nonwoven textile). The anti-biofilm activity of AgNPs and the ability of AgNPs to transfer toxic properties into the polymer matrix were proved.

## 1. Introduction

Nanotechnology is one of the most exciting and fast-moving areas at present in science and research. Naturally occurring nanostructures are present around us in nature, in plants, and also in animals. Thanks to this, scientists can now produce nanoparticles by many methods. The nanoparticles can be synthesized in two main ways: “top–down” and “bottom–up” [1]. Thanks to these methods, we can obtain nanomaterials with the required shapes, sizes, and special properties. In the “top–down” method, various procedures such as milling, etching, or sputtering are used; in the “bottom–up” method, reducing agents, stabilizing agents, and solvents are used for the synthesis of nanoparticles [2]. The “bottom–up” methods also include chemical reduction and the so-called green synthesis methods (biological methods). Often, the chemical synthesis technique results in the adsorption of some harmful compounds on the surface of the nanoparticle, which might have a detrimental effect on medical applications [3]. Thanks to their ecological advantages, the biosynthesis of nanoparticles has recently come to the forefront [4]. Metal nanoparticles can be easily, ecologically, and cost-effectively created using extracts from plants, fungi, microorganisms, yeast, algae, etc. Green methods are more popular mainly because of the absence of toxic solvents that are difficult to degrade. Therefore, green synthesis appears to be a good alternative to conventional methods of AgNP synthesis, which are widely used in various industries, such as medicine, biology, electrical technology, and others.

The significance of nanoparticles is that they have different properties compared with the bulk material of the same class. A nanoparticle is a particle that ranges between 1 and 100 nanometers (1–100 nm) [5]. For the properties and use of nanoparticles, their size and shape are decisive. 

The worldwide alarming multi-resistance to antibiotics creates the need for effective strategies to fight against bacteria. AgNPs represent a new approach to fighting and eliminating microorganisms. AgNPs have antibacterial, antifungal, anti-inflammatory, antiviral, anti-angiogenic, and anti-cancer activities. Currently, much of the research synthesizes AgNPs by various methods and tests their anti-biofilm activity on various gram+ and gram- bacteria, various viruses and fungi, and others [6].

They also have excellent optical, physical, chemical, and electrical properties [7]. There are many ways to prepare nanoparticles using green synthesis techniques. For example, Kumar et al. described a biosynthesis using lavender leaf extract utilizing 4-nitrophenol and H_2_O_2_ and NaBH_4_ in addition to AgNO_3_ [8]. Hasanin et al. also successfully prepared silver nanoparticles using an extract from the overground parts of *Lavandula coronopifolia* and AgNO_3_ precursor [9,10].

The main goal of incorporating nanoparticles into the polymer matrix is to expand the area of possible applications of colloidal AgNPs and to develop a nanocomposite with better properties that depend on the properties of the filler (NPs). A polymer nanocomposite (PNC) is a complex of the matrix and the filler. PNCs represent a new class of materials with wide applications [11,12]. Polymers such as nylon, polyurethane (PU), polybenzimidazole (PBI), polycarbonate (PC), polyacrylonitrile (PAN), polyvinyl alcohol (PVA), poly lactic acid (PLA), polyvinylpyrrolidone (PVP), poly-methacrylate (PMMA), polyethylene oxide (PEG), polyaniline (PANI), polystyrene (PS), collagen, chitosan, chitin, cellulose, etc., can be used as matrixes [13].

There are many methods for the production of nanocomposites; the basic methods are “in situ” and “ex situ”. The difference between the “in situ” and “ex situ” methods is the method of adding the secondary phase. With the “in situ” method, nanoparticles are formed directly in the matrix during the production of the composite. With the “ex situ” method, the secondary phase is prefabricated separately and then added to the polymer matrix. Each of these methods has its advantages and disadvantages. The advantage of the “ex situ” method is due to the fact that we know the size and shape of the added nanoparticles in advance.

Methods such as dip-coating, spin-coating, casting, or electrospinning enable easy preparation of the final product of nanocomposites (thin layers, nanofibers, etc.) [13]. The electrospinning process is one of the most cost-effective and efficient techniques for the production of micro- and nano-diameter fibers [14,15].

Silver nanoparticles opened a new era of material in the nanoscale. The application of nanoparticles is very wide thanks to their small size and large surface. 

They are characterized by unique properties such as physical, chemical, optical, catalytic, electrical, thermal, high electrical conductivity properties, and many others. Due to their special properties, they are used for several applications, including antibacterial agents, in industrial, household, medical, and consumer products, and as coatings for medical devices, optical sensors, and cosmetics [7]. They are also used in the pharmaceutical industry, the food industry, diagnostics, orthopedics, and drug delivery. Recently, AgNPs have been widely used in many textiles, offices, wound bandages, and biomedical devices. 

They can replace the antibiotics that are used in the treatment of artificial implants and that cause a dependence in their users. Silver was used in the past, e.g., for food conservation and wound healing. Silver nanoparticles not only have the same properties as bulk silver they can also be designed to be selective against bacteria and pathogens and friendly to skin cells [6]. 

The aim of this work was to prepare a polymer nanocomposite doped with AgNPs. Conventional methods of producing nanomaterials use expensive chemical and physical processes that utilize materials with potential risks, such as toxicity, cytotoxicity, and carcinogenicity. Nanoparticles were fabricated by green synthesis, which is ecological, inexpensive, time-saving, and effective. Silver is known for its excellent antibacterial properties, and the goal was to transfer these properties to a non-toxic PVA matrix. We assumed that the incorporation of silver nanoparticles into the PVA matrix would change the properties of the polymer. This should increase the application possibilities of PVA.

## 2. Materials and Methods

### 2.1. Materials

Lavender leaves were gathered in a local garden in Košice (Slovakia) during the flowering period (July). Silver nitrate (AgNO_3_, 99.8%) was purchased through Mikrochem Ltd., Pezinok, Slovakia, as a silver precursor. Deionized water was used for the preparation and dilution of all solutions. Polyvinyl alcohol (PVA) (M.W. approx. 146,000–186,000, purchased through Mikrochem Ltd., Pezinok, Slovakia) was used as a matrix for the production of polymer matrix composite doped by AgNPs.

### 2.2. Leaves Extract Production

**Figure 1:**
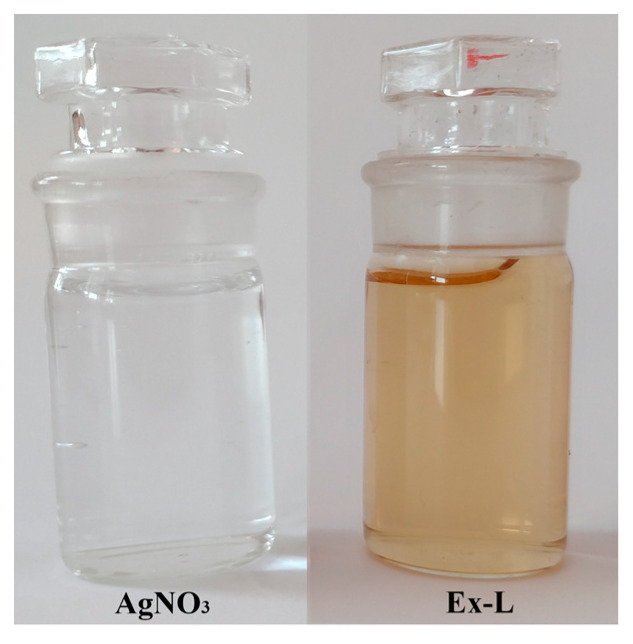
AgNO_3_ solution and extract of dried lavender leaves (Ex-L).

The extract was prepared from the dried leaves of lavender. Dried leaves (2.74 g) were washed twice with deionized water and ground in a mortar, and then 100 mL of deionized water was added. Subsequently, the mixture was heated in a water bath at a temperature ranging from 70 to 80 °C for 10 min. The solid part was separated from the liquid with filter paper. The filtered solution was subsequently centrifuged at 9000 rpm for 30 min. The prepared extract was stored in a fridge for further experiments. Figure 1 shows the AgNO_3_ stock solution and extract prepared from dried lavender leaves (Ex-L).

### 2.3. Preparation of AgNPs

First, 100 mL of AgNO_3_ stock solution with a concentration of c = 50 mg/L (0.46 mM) was poured into an Erlenmeyer flask. The AgNO_3_ solution was heated at a constant stirring to a temperature of 80 °C. After reaching the temperature, 20 mL of extract (Ex-L) was slowly poured into the flask. The sample was stored at a temperature of 80 °C for 20 min. Subsequently, after cooling, the solution was subjected to UV-vis analysis and stored under different conditions to monitor AgNPs’ long-term stability.

### 2.4. Long-Term Stability of AgNPs

The stability of nanoparticles was monitored under two different conditions: at room temperature (light conditions: light:dark = 12:12 h) and in the refrigerator. For this purpose, the prepared colloidal solution of AgNPs (120 mL) was divided into two 60 mL Erlenmeyer flasks. The solutions were labeled as follows:

- L-Cold–AgNPs colloid stored in the refrigerator (~5 °C);
- L-RT–AgNPs colloid stored at room temperature (~25 °C).

### 2.5. Preparation of Polymer Nanocomposite

We used an “ex situ” method to produce the nanocomposite (PVA-AgNPs). The PVA polymer was used as a matrix and biologically pre-prepared AgNPs as a secondary phase. For 8% solution of PVA-AgNPs, 4 g of PVA and 46 mL of AgNPs colloid were mixed and stirred at 80 °C for 1 h to achieve a uniform distribution of AgNPs.

After the incorporation of AgNPs into PVA, the solution was spun by needleless electrospinning techniques and cast as PVA-AgNPs composite in a thin film. The applied voltage at electrospinning was 82 kV and the distance between the spinning and collector electrodes was 150 mm.

### 2.6. Characterization of AgNPs and PVA-AgNPs Composites

The composition of the extract was analyzed using an FTIR spectrometer. The infrared spectra were recorded on a Bruker Tensor 27 FTIR spectrometer equipped with a DTGS KBr detector. For each sample, 64 scans were measured in the 4000–400 cm^−1^ spectral range in the abs mode with a resolution of 4 cm^−1^. The KBr pressed-disc technique was used for preparing a solid sample for routine scanning of the spectra. Samples of approximately 0.1 mg were dispersed in 150 mg of KBr to achieve optimal spectra in the regions 4000–400 cm^−1^. The diameter of the pellets pressed from samples was 13 mm. 

Prepared AgNPs colloid solutions were analyzed by UV-Vis Spectrophotometer (UNICAM UV-vis Spectrophotometer UV4). The samples were measured on the 1st, 3rd, 7th, 10th, 14th, and 21st day in the wavelength range of 350–700 nm. As a control, a solution of H_2_O extract in a ratio of 5:1 was used.

The size and morphology of the nanoparticles were studied by means of TEM (JEOL model JEM-2000FX, an accelerating voltage of 200 kV). The image analysis (ImageJ software, 1.53t) was used for the analysis of Ag nanoparticles’ size distribution. The morphology of AgNPs and the presence and distribution of nanoparticles in PVA-AgNP composite thin films and fibers were observed by scanning electron microscopy SEM/FIB (SEM/FIB ZEISS-AURIGA Compact). The phase composition of the samples was analyzed using X-ray diffraction analysis (XRD, ZEISS, Oberkochen, Germany).

### 2.7. The Anti-Biofilm Activity

The anti-biofilm activity of AgNPs was evaluated using the standard disk-diffusion method described by Kavita et al., with some modifications [16,17]. Milieu Bristol agar plates in Petri dishes were in a sterile box inoculated with green algae *Chlorella kessleri*. The sterile swabs were plated on agar. Subsequently, 15 µL of both Ex-L and colloid of AgNPs were dropped on sterile swabs. These prepared samples were incubated at room temperature in dark:light conditions, 12:12 h, respectively. Three replicate samples were tested for each condition. The control tests with AgNO_3_ (0.46 mM) were also provided. The presence and size of the inhibition zone were checked after 14 days of growth [18]. 

Samples in the form of discs were cut out of the PVA-AgNPs composite fibers and thin film and put directly on agar plates inoculated by green algae *Chlorella kessleri*. The anti-biofilm activity of produced polymer composites was observed at the same conditions as in the previous samples.

## 3. Results and Discussion

### 3.1. FTIR Analysis

**Figure 2:**
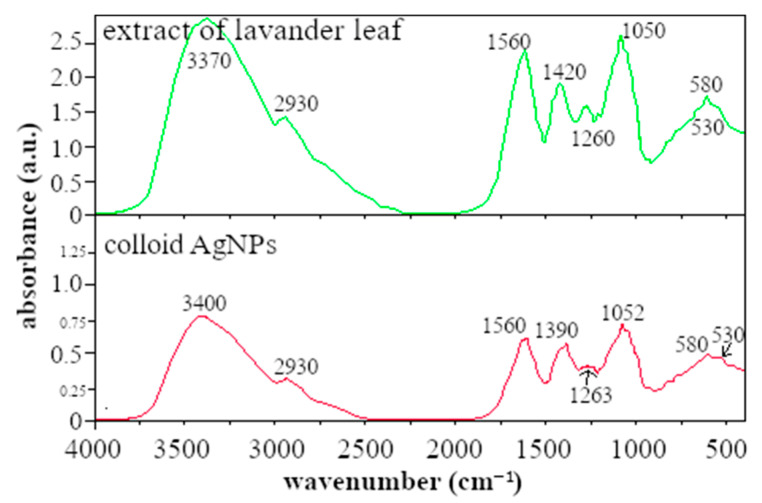
Comparison of the IR spectra of dried lavender leaf extract and AgNPs colloid prepared by the dry lavender leaf extract.

A comparison of the IR spectra of dried lavender leaf extract and Ag colloid prepared using dry lavender leaf extract is shown in Figure 2. The IR spectra of lavender leaf extract and AgNPs colloid showed a high absorption band in the spectral region of 2800–3400 cm^−1^, which can be attributed to the bounded hydroxyl (-OH) groups of alcohols or phenols [19,20,21]. Previous studies suggested that these bonds could be due to the hydroxyl groups present in proteins, enzymes, or polysaccharides found in the lavender leaf extract [22]. A smaller band at a wavelength of around 2930 cm^−1^ is caused by the presence of -CH valence bonds of the alkanes [23].

The absorption band at 1560 cm^−1^ observed in the extract and AgNPs colloid sample may be due to amide I and amide II formed through carbonyl and -N-H stretch vibrations in the amide linkages of proteins, respectively. The 1420 cm^−1^ band corresponds to the C-O-H group. Absorption bands around 1263 cm^−1^ and 1052 cm^−1^ can be attributed to the C-O valence vibrations of the ester groups [24,25] or to the C-N valence vibrations of the amine groups, respectively. The peak at ∼1260 cm^−1^ is also reported in ref. [26]; the authors assign this peak to the C-O functional group, as we do. The authors assumed that the C-O group is involved in the synthesis of AgNPs and therefore the peak decreases. 

Ahmed et al., in their work, described that the presence of long-chain hydrocarbons and polar ester linkage supports better stabilization [27]. The lower bands at 586 and 530 cm^−1^ can be attributed to the alkyl halides such as C-Cl stretching found in anthocyanin flavonoids [23], which are present in the leaf extract as well as in the AgNP colloid. Lavender also contains gallic acid (phenolic flavonoids). 

As can be seen from the FTIR analysis, the leaf extract has a very complex composition; therefore, it is difficult to determine which components are responsible for the reduction of Ag^+^ ions to silver nanoparticles and which for the stabilization. Based on the other authors’ results and our observations, free groups present in proteins and flavonoids found in leaf extracts are responsible for the reduction of Ag^+^ ions. The C=O groups form the protective layer band and act as the capping agent which stabilizes and prevents aggregation of AgNPs in polar solution [23]. The negative charge of the compounds present in the extracts provides electrostatic barriers around the nanoparticles, contributing to AgNPs’ stability.

### 3.2. UV-Vis Analysis

The production of nanoparticles was observed almost immediately after mixing the reactants; the solution turned light yellow, which confirmed the synthesis of AgNPs. After 20 min, the color of the solution changed from light yellow to brown, indicating an increase in the concentration of nanoparticles in the solution.

The presence of AgNPs in the solution was confirmed by UV-Vis spectrophotometry. UV-Vis analysis is a simple, reliable method to prove the presence of nanoparticles in solutions. Based on the shape and location of the SPR band, the size, size distribution, and shape of the nanoparticles can be approximately determined. A colloidal solution of spherical silver nanoparticles is typical of the maximum surface plasmon resonance (SPR) band in the wavelength interval of 350–450 nm [8,28]. Solutions that do not contain nanoparticles (input solutions and control solutions) do not show any peaks; the SPR curves of such solutions are reported by other authors in their papers [29,30]. After synthesizing spherical nanoparticles, they observed the formation of peaks at ~450 nm.

The strong, slim peak of the SPR band with an absorption maximum (ABSmax) at a wavelength of 424 nm was measured. Based on this, it is possible to assume the formation of spherical silver nanoparticles. The same conclusion from the SPR band for silver nanoparticles prepared by green synthesis was reported by Kumar et al. [31].

**Figure 3:**
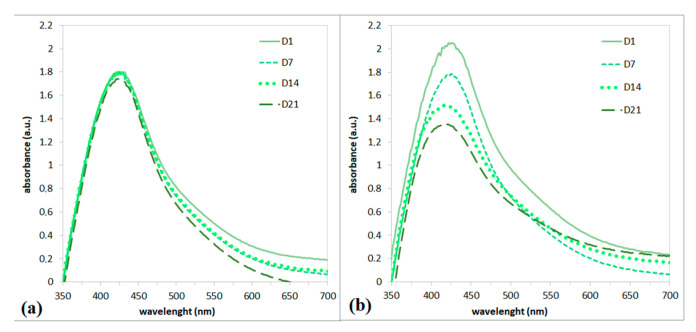
UV-Vis spectrum of AgNPs prepared using Ex-L stored in the cold (**a**) and stored at room temperature (**b**).

An important property of nanoparticle colloids is their stability, which depends on several factors, such as the presence of stabilizing substances in the extracts and storage conditions. To evaluate the long-term stability, the prepared AgNP colloid solution was divided into two flasks where one of them was stored in the refrigerator (L-Cold) and the second at room temperature (L-RT). Figure 3a,b show the SPR bands of both L-Cold and L-RT colloids measured on the 1st; 7th; 14th; and 21st day. Figure 3a shows the SPR bands of the colloidal solution L-Cold. Based on the shape and position of SPR bands which are slim, symmetrical, and stable, it can be concluded that the nanoparticles were in a narrow size interval distribution and stable during the whole experiment.

Figure 3b shows a comparison of colloidal solutions stored at room temperature. It is well known that silver nanoparticles are sensitive to light. It is clear that the solution of silver nanoparticles stored at RT was not stable. A decrease in ABS_max_ was observed over time, indicating degradation by dissolution or agglomeration of the nanoparticles. At the end of the experiment, sediment was also observed at the bottom of the flask; therefore, the agglomeration of nanoparticles can be assumed. It is clear that storage conditions significantly affect the stability of AgNP colloids. Cold storage provides better stability. Based on the stability results, cold-stored nanoparticles were used for further experiments.

### 3.3. TEM and SEM Analyses

**Figure 4:**
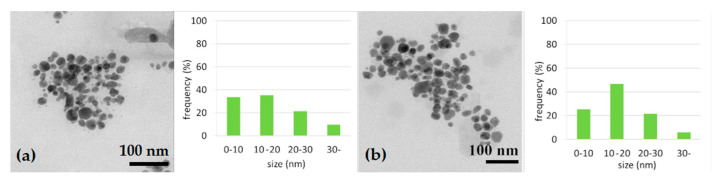
TEM image of AgNPs and size distribution of nanoparticles on D0 (**a**) and on D21 (**b**).

The morphology and size of AgNPs were determined using TEM analysis. Figure 4a,b show TEM images of silver nanoparticles and their size distribution on day 0 and day 21. TEM analysis of both samples confirmed spherical nanoparticles in similar size intervals; 75% of them reached up to an average size of 20 nm. This result confirms that leaf extract can form stable, spherical nanoparticles of up to 20 nm.

**Figure 5:**
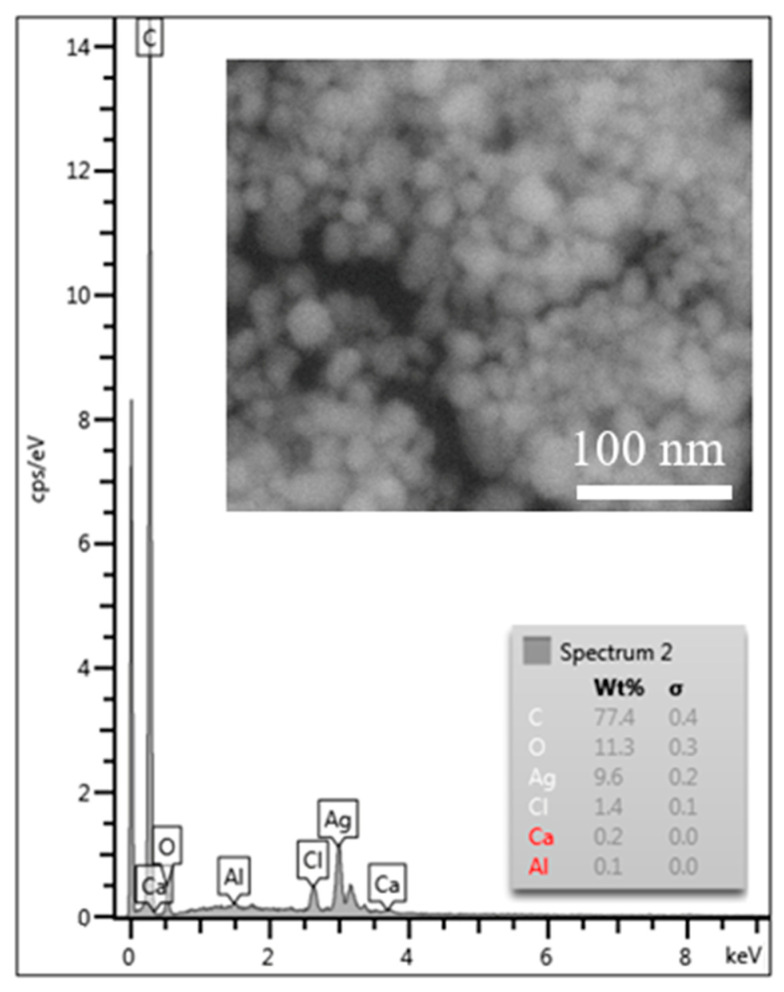
EDX analysis of AgNPs. The inner picture shows SEM microstructure of AgNPs prepared by Ex-L.

Analysis by scanning electron microscopy, Figure 5, confirmed the presence of uniform, near-spherical AgNPs. EDX analysis, Figure 5, confirmed silver nanoparticles and demonstrated the presence of signals of accompanying elements such as C, Cl, O, and Ca. These elements probably came from the biological component of the extract, which could be removed from the sample by thoroughly purifying it in ethanol.

### 3.4. Polymer Matrix Nanocomposite (Nanofibers, Nanofilm)

**Figure 6:**
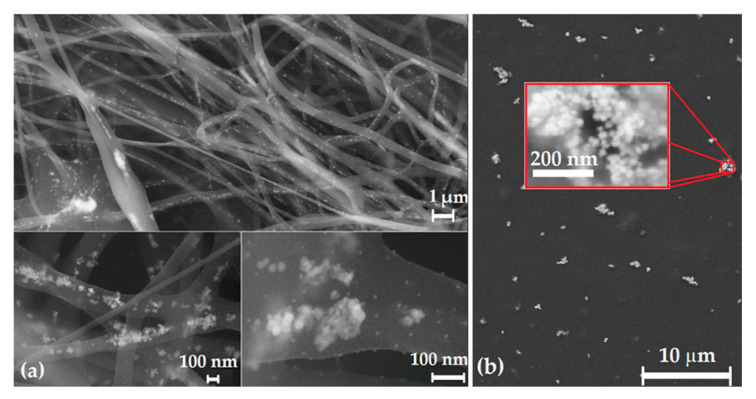
SEM image of AgNP clusters in PVA-AgNPs nanofibers (**a**) and thin film (**b**).

To prepare the polymer matrix composite, PVA was used as a matrix and prepared AgNPs as a second phase. Subsequently, the thin layers and fibers were prepared. The presence of AgNPs in the thin layer and fibers of the PVA-AgNP composite was observed by SEM. The results are shown in Figure 6. It is obvious that the nanoparticles were not uniformly distributed along the length of each fiber (Figure 6a). In the details of the nanofiber, we can observe the morphology and distribution of the nanoparticles, which are spherical in shape and are dispersed individually or in clusters. We can also observe that nanoparticles are not only inside the fibers but also on the fibers’ surface. The same non-uniform distribution of nanoparticles can be observed in the thin film (Figure 6b). The nanoparticles mostly form clusters.

It is obvious that the used method of PVA-AgNP composite preparation did not result in uniform distribution of nanoparticles. The clusters are evident in both composite samples. Better distribution of AgNPs in the matrix can be secured by applying ultrasonic, more intensive, and longer stirring, as well as increasing the solution temperature during stirring. Another possibility is to prepare the solution with lower density, with subsequent evaporation to the required 8% solution.

It is known from several studies that by using ultrasound it is possible to break agglomerates and thus ensure a better distribution of particles [32,33]. Sumitomo, et al., compared the effect of ultrasonication and mechanical stirring on particle dispersion. They optimized the ultrasonication conditions that ensure uniform nanoparticle distribution [34]. Based on these results we suppose that the nanoparticle agglomerates can be broken by ultrasonication, which improves the size distribution of nanoparticles in the matrix.

### 3.5. Anti-Biofilm Activity 

The antibacterial activity of silver nanoparticles was observed using the green algae *Chlorella kessleri* with a modified disc diffusion method. We inoculated algae on sterile Petri dishes (PD) with agar in a sterile box and sterile swabs were set on agar. On the swabs, 15 µL of AgNPs colloid, and Ex-L were dripped. Ex-L was used as a control. Additionally, the PVA-AgNPs composite thin film and fibers were tested. The anti-biofilm activity was monitored for 14 days.

**Figure 7:**
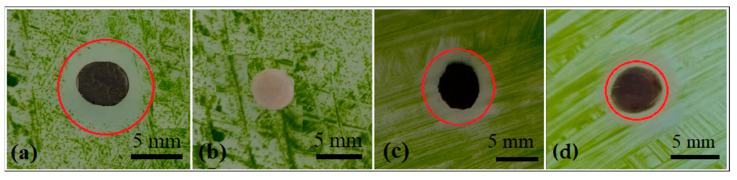
Anti-biofilm activity of AgNPs (**a**), anti-biofilm activity of Ex-L (**b**), anti-biofilm activity of AgNP nanofibers (**c**), and thin film (**d**).

Ex-L, Figure 7b, did not show any inhibition zones during the 14 days. An inhibition zone of 9.3 mm and 8.9 mm was observed for AgNP colloid and AgNPs incorporated in fiber, respectively, Figure 7a,c. PVA-AgNPs composite thin film did not show distinctive anti-biofilm activity; a 1–2 mm inhibition zone was observed (Figure 7d). 

The antibacterial properties of AgNPs depend on the size of the nanoparticles; it is assumed that smaller silver nanoparticles pass through the cell wall more easily and, in addition, have larger reaction surfaces and therefore should show better antibacterial activity. Szerencsés et al. confirmed that the size of AgNPs affects their anti-biofilm activity. They tested three different sizes of AgNPs: ~7.0 nm; ~21 nm; and ~50 nm. The antifungal activity test revealed that silver nanoparticles with the smallest size were the most effective [35].

In our case, 75% of nanoparticles had a diameter of up to 20 nm, and the maximum measured diameter was 50 nm. According to the results, where the inhibition zone of 9.3 mm was measured, the presence of 75% of small nanoparticles ensures very good anti-biofilm activity (Figure 7a).

On the other side, the extract was not toxic at all; we suggested that the lavender does not contain toxic compounds, Figure 7b. Pei Lin et al. found the same results as us. They tested the antibacterial properties of silver nanoparticles and the lavender extract showed no inhibition zone [36].

The fibers also showed a significant inhibition zone, Figure 7c. The thin film showed only a weak inhibition zone, Figure 7d. The reason for better antibiofilm activity, in the case of fibers, is the fact that nanoparticles in the fibers were placed both inside the fibers and on the surface. In the case of thin film, the nanoparticles were surrounded by a PVA matrix, preventing their direct contact with the algae cells.

## 4. Conclusions

The aim of the work was to prepare silver nanoparticles by a green method and incorporate them into the polymer matrix. We proved that the extract prepared from lavender leaves is able to synthesize AgNPs. Of the prepared nanoparticles, 75% reached up to an average size of 20 nm. It is obvious from the results that the nanoparticles were stable for up to 21 days, constituting long-term stability. Prepared AgNPs were successfully incorporated into the polymer matrix by an ex situ method. A PVA-AgNP composite was used for nanofiber and thin-layer production. After the incorporation of AgNPs into a non-toxic matrix, the anti-biofilm activity (on algae *Ch. kessleri*) was proved. The inhibition zones were 9.3 mm for the AgNP colloidal solution, 8.9 mm for the fibers, and 5.4 mm for the thin film. It is obvious that silver nanoparticles prepared using lavender leaf extract in the form of a colloidal solution show high anti-biofilm activity. The colloidal solution prepared this way has a wide range of uses. The produced nanofibers and thin layers doped with AgNPs can be widely used in several industries. Such composites protect against microorganisms in aqueous and humid environments, such as boats, food, fashion, and in the medical industry.

## Data Availability

Not applicable.
